# Can fat infiltration in the multifidus muscle be a predictor of postoperative symptoms and complications in patients undergoing lumbar fusion for degenerative lumbar spinal stenosis? A case–control study

**DOI:** 10.1186/s13018-022-03186-2

**Published:** 2022-05-26

**Authors:** Gengyu Han, Da Zou, Xinhang Li, Shuquan Zhang, Zhenxu Li, Siyu Zhou, Wei Li, Zhuoran Sun, Weishi Li

**Affiliations:** 1 Department of Orthopaedics, Peking University Third Hospital, No. 49 NorthGarden Road, Haidian District, Beijing, 100191 China; 2 Engineering Research Center of Bone and Joint Precision Medicine, Ministry of Education, Beijing, China; 3 Beijing Key Laboratory of Spinal Disease Research, Beijing, China

**Keywords:** Clinical outcome, Fat infiltration, Lumbar spinal stenosis, Multifidus

## Abstract

**Purpose:**

This study aimed to explore whether 25% as the cutoff value of fat infiltration (FI) in multifidus (MF) could be a predictor of clinical outcomes of lumbar spinal stenosis (LSS) patients.

**Methods:**

A total of 461 patients undergoing posterior lumbar interbody fusion for LSS with 1-year follow-up were identified. After sex- and age-match, 160 pairs of patients were divided into a FI < 25% group and a FI ≥ 25% group according to FI of MF at L4 on preoperative magnetic resonance imaging. Patient-reported outcomes including the visual analog scale scores (VAS) for back pain and leg pain and the Oswestry disability index (ODI) scores were evaluated. Bone nonunion and screw loosening were evaluated by dynamic X-ray.

**Results:**

After matching, there was no significant difference in age, sex, body mass index, fusion to S1, number of fusion levels, osteoporosis, spondylolisthesis, smoking and diabetes. FI ≥ 25% group had significantly higher VAS for back pain, VAS for leg pain and ODI than FI < 25% group at 1-year follow-up. However, there was no significant difference in the change of them from baseline to 1-year follow-up between the two groups. In light of complications, FI ≥ 25% group had a significantly higher rate of bone nonunion than FI < 25% group, whereas there was no significant difference of screw loosening rates between the two groups.

**Conclusion:**

MF FI might be a pragmatic cutoff value to predict bone nonunion in LSS patients, but it has little predictive value on screw loosening and postoperative improvement of symptoms.

**Supplementary Information:**

The online version contains supplementary material available at 10.1186/s13018-022-03186-2.

## Background

Lumbar spinal stenosis (LSS) has been one of the most common causes of surgery with a growƒing burden in an aging population [1]. Although surgical treatment has succeeded in relieving pains of patients, several complications and unabated pain still occur after surgery in LSS patients [2].

Studies have revealed that the paraspinal muscle degeneration, a universal phenomenon among old people, is implicated in multiple degenerative lumbar pathologies [3–5]. Lumbar multifidus (MF) is one of paravertebral muscles, playing an important role in stabilizing lumbar spine. Fat infiltration (FI) is a frequently used indicator to assess the degeneration of muscle composition [5].

Currently, the predictive value of paraspinal muscle morphometry for several surgical disciplines inclusive of metastatic disease, trauma and fracture on image examination is being unearthed [6–8]. Degeneration of MF has been proved to be related to several lumbar diseases including LSS [9–11]. However, the cutoff value of FI has not been established, leading to a poor use in clinical application. Liu et al. attempted to divide patients into two groups by using 25% as the cutoff value. They finally suggested that FI in MF could be a potential predictor of improvement of functional status and symptoms in LSS patient [12]. However, the sample size of the study was relatively small. Besides, the association between FI and surgical complications has not been investigated.

Therefore, the purpose of this study was to demonstrate whether 25% as the cutoff value of MF FI could be a predictor of postoperative symptoms and complications of LSS patients.

## Materials and methods

This retrospective study was approved by the institutional review board, with the requirement for informed consent being waived (M2020496). Hospitalized patients undergoing posterior lumbar interbody fusion for LSS between July 2011 and December 2016 were reviewed. Inclusion criteria were as follows: (1) aged ≥ more than 40 years, (2) underwent lumbar magnetic resonance imaging (MRI) and lumbar computed tomography (CT) within 3 months before the index surgery, (3) underwent follow-up of ≥ 12 months. Exclusion criteria were as follows: (1) previous spinal surgery, (2) patients with bone tumor, ankylosing spondylitis, diffuse idiopathic skeletal hyperostosis, rheumatoid arthritis, tuberculosis or secondary osteoporosis, (3) previous or current hormone therapy and (4) patients with spondylolisthesis (> grade 1) or scoliosis (> 10°). A total of 461 patients were identified.

### Patient-reported outcomes

Patient-reported outcomes, including the visual analog scale scores (VAS) for back pain and leg pain and the Oswestry disability index (ODI) scores ranging from 0 to 100, with the highest score indicating the worst disability, were evaluated at baseline and 1-year follow-up. Clinically significant improvement (CSI) in each domain of interest was defined as improvement rate ≥ 50% for VAS-back, improvement rate ≥ 50% for VAS-leg or improvement rate ≥ 40% for ODI [12].

### Radiographic evaluation

**Fig. 1:**
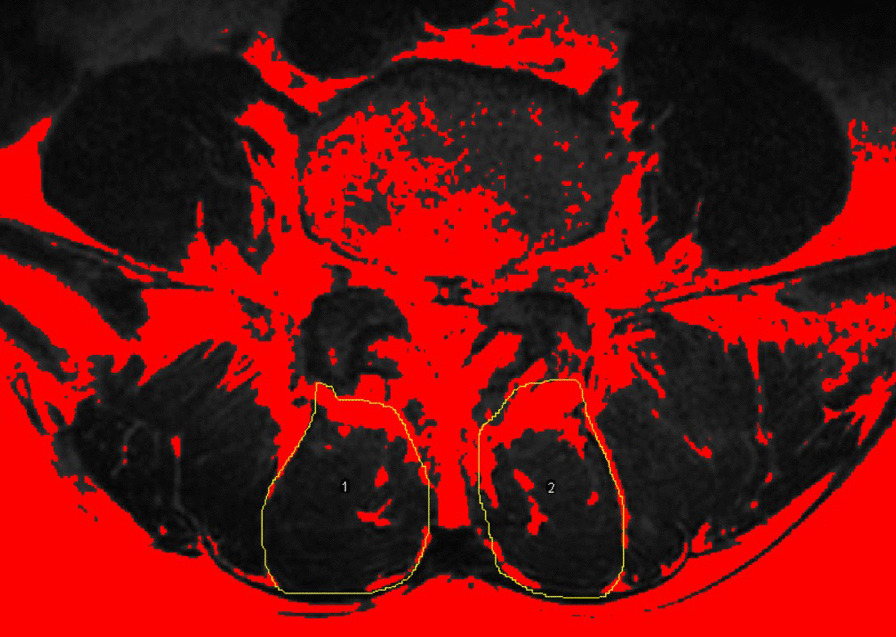
Measurements of paraspinal muscular parameters on axial T2-weighted MRI (a 55-year-old man). Regions of multifidus at L4 level were outlined by yellow lines. Thresholding technique to highlight fatty area (red area)

All enrolled patients had undergone preoperative MRI of lumbar area with Signa HDxt 3.0 T (General Electric Company). The fatty infiltration (FI) of bilateral MF was evaluated at the superior end plate level of L4 from T2-weighted images using the thresholding technique in ImageJ software version 1.5 (National Institutes of Health, Bethesda, Maryland, USA, Fig. 1) [13, 14]. To test the reliability, all muscular parameters of 20 patients were randomly selected and were measured by two observers independently. After 3 weeks, the same measurements were performed by one observer. The ICCs for both intra-rater and inter-rater reliability were > 0.8.

Segmental fusion status and screw loosening were evaluated by dynamic X-ray after 1-year follow-up by two observers independently. We defined the bone nonunion as (1) there was no continued bone fusion mass at any fusion segment and (2) any motion (greater than 3 mm or 3°) on flexion/extension plain radiographs [15]. Screw loosening was defined when a 1-mm or wider circumferential radiolucent line around the pedicle screw was confirmed on spine radiograph at 1-year follow-up [16]. Observers were blinded to clinical information, and the evaluation of complications was separated from muscular measurements.

### Statistical analyses

All enrolled patients were divided into a FI < 25% group and a FI ≥ 25% group according to FI of MF at L4. To reduce the bias, we selected patients with FI ≥ 25% to match to patients with FI < 25% in a 1:1 manner according to age (the difference was less than 3 years) and sex. As a result, 320 patients were selected in this study (Additional file 1: Fig. S1).

Previous study has reported that the mean value of MF FI in patients with lumbar diseases was close to 25% [17]. Besides, Peng et al. have revealed that MF FI in 70–79 years group was 25.84% [18]. Moreover, Liu et al. have divided patients into FI < 25% and FI ≥ 25% groups to investigate the relationship between MF FI and surgical prognosis [12]. In terms of level selection, increased FI of paraspinals has been found particularly at L4/5 [19].On the other hand, Crawford et al. have shown that the fat content at L4 best represents that of the entire lumbar region in healthy participants, providing a time-efficient capture of lumbar paravertebral FI [20]. Therefore, in the current study, we considered an FI of MF at L4 ≥ 25% as the exposure and defined it as significant fatty infiltration versus an FI < 25% as insignificant fatty infiltration [12].

The age- and sex-matching process was performed with the case–control matching (CCM) function of SPSS. The Mann–Whitney U test or ANOVA (for continuous data) and Chi-square test (for categorical data) were conducted to determine the statistical difference. Intraclass correlation coefficient was calculated to test the intra- and inter-rater reliability. Statistical significance was set at *P* value < 0.05. All statistical analyses were performed using SPSS 22.0 (IBM Corp).

## Results

A total of 461 patients have been received in our study, with an average age of 60 years. The whole patients have been divided into FI < 25% group (n = 235) and FI ≥ 25% group (n = 226). Before CCM, the average age was 58.0 and 62.0 years in FI < 25% and FI ≥ 25% groups, respectively (*P* < 0.001). There was also a significant difference in sex and osteoporosis between two groups (both *P* < 0.001). However, VAS for back pain (5.1 vs. 5.4, *P* = 0.268), VAS for leg pain (5.8 vs. 6.1, *P* = 0.186) and ODI (41.0 vs. 41.5, *P* = 0.591) had no significantly difference between the two groups. The data are shown in Table 1.

**Table 1:** Comparisons of clinical data at baseline between MF FI ≥ 25% and FI < 25% groups in complete cohort

Variable	FI ≥ 25% (n = 226)	FI < 25%(n = 235)	*P* value
Age (years)	62.0 ± 7.3	58.0 ± 7.4	< 0.001**
Sex (male/female)	69:157	116:119	< 0.001**
BMI (kg/m^2^)	26.0 ± 3.4	26.1 ± 3.3	0.815
Fusion to S1 (no: yes)	134:92	145:89	0.533
Number of fusion levels	1.9 ± 0.9	1.9 ± 0.9	0.937
Osteoporosis (no: yes)	145:79	185:47	< 0.001**
Spondylolisthesis (no: yes)	123:67	121:40	0.035*
Smoking (no: yes)	194:32	200:35	0.823
Diabetes (no: yes)	189:37	201:34	0.571
MF FI (%)	36.3 ± 9.2	18.3 ± 4.7	< 0.001**
*Baseline*			
VAS for back pain	5.4 ± 2.0	5.1 ± 2.2	0.268
VAS for leg pain	6.1 ± 2.1	5.8 ± 2.3	0.186
ODI	41.5 ± 18.8	41.0 ± 20.3	0.591

The numbers in bold represented that there was significant difference between the two groups

*P < 0.05, **P < 0.01

Based on CCM, we successfully matched 160 pairs of patients. After CCM, there was no significant difference in age, sex, body mass index (BMI), fusion to S1, number of fusion levels, osteoporosis, spondylolisthesis, smoking and diabetes (all *P* > 0.15, Table 2). For the baseline data, FI ≥ 25% group had slightly higher VAS for back pain, VAS for leg pain and ODI than FI < 25% group without significant difference (all *P* > 0.05, Table 2).

**Table 2:** Comparisons of clinical data and patient-reported outcomes after case–control matching

Variable	FI > 25% (n = 160)	FI < 25% (n = 160)	*P* value
Age (years)	60.7 ± 7.0	59.9 ± 6.7	0.231
Sex (female/male)	56:104	56:104	1.000
BMI (kg/m2)	25.9 ± 3.6	26.0 ± 3.4	0.929
Fusion to S1 (no:yes)	93:67	100:60	0.424
Number of fusion levels	1.9 ± 0.9	1.9 ± 0.8	0.581
Osteoporosis (no:yes)	113:47	115:45	0.805
Spondylolisthesis (no:yes)	88:48	90:36	0.244
Smoking (no:yes)	132:28	141:19	0.155
Diabetes (no:yes)	137:23	138:22	0.872
MF FI (%)	36.6 ± 9.7	18.9 ± 4.5	< 0.001**
*Baseline*			
VAS for back pain	5.4 ± 2.0	5.3 ± 2.3	0.819
VAS for leg pain	6.0 ± 2.1	5.9 ± 2.4	0.931
ODI	41.3 ± 18.6	40.6 ± 19.9	0.684

The numbers in bold represented that there was significant difference between the two groups

*P < 0.05, **P < 0.01

At 1-year follow-up, FI ≥ 25% group had significantly higher VAS for back pain, VAS for leg pain and ODI than FI < 25% group (2.8 vs. 3.2, *P* = 0.017; 2.3 vs. 2.8, *P* = 0.039; 17.1 vs. 21.7, *P* = 0.010, respectively; Table 3). However, there was no significant difference in the change of them from baseline to 1-year follow-up between FI < 25% and FI ≥ 25% groups (all *P* > 0.05, Table 3). The proportion achieving CSI with respect to VAS for back pain in FI < 25% group was higher than that in FI ≥ 25% group , but not statistically significant (52.2% vs 48.9%, *P* = 0.913). Moreover, no significant differences in the proportion achieving CSI of VAS for leg pain (70.0% vs. 58.0%, *P* = 0.548) and ODI (61.8% vs. 58.5%, *P* = 0.879) were found between the two groups (Table 3).

**Table 3:** Comparisons of the Improvement of Patient-Reported Outcomes At 1-year follow-up After Case–Control Matching

Variable	FI > 25% (n = 160)	FI < 25% (n = 160)	*P* value
*At 1-Year Follow-Up*			
VAS for back pain	3.2 ± 2.3	2.8 ± 2.2	0.017*
VAS for leg pain	2.8 ± 2.4	2.3 ± 2.5	0.039*
ODI	21.7 ± 15.1	17.1 ± 14.1	0.010*
*Change*			
Change of VAS for back pain	2.5 ± 2.7	2.5 ± 2.9	0.527
Change of VAS for leg pain	3.2 ± 3.0	3.7 ± 3.5	0.212
Change of ODI	17.9 ± 17.7	21.0 ± 23.0	0.549
*Rates of CSI*			
Improvement rate of VAS for back pain ≥ 50%	69:66	55:60	0.913
Improvement rate of VAS for leg pain ≥ 50%	55:76	34:79	0.548
Improvement rate of ODI ≥ 40%	56:79	47:76	0.879

The numbers in bold represented that there was significant difference between the two groups

*P < 0.05, **P < 0.01

In light of complications, bone nonunion occurred in 12 patients in FI < 25% group, which was significantly lower than 40 patients in FI ≥ 25% group (*P* < 0.001; Table 4). Screw loosening had a higher ratio to occur in FI ≥ 25% group than FI < 25% group, whereas there was no significant difference in screw loosening rates between the two groups (41 vs 55, *P* = 0.073; Table 4). Further, we performed a subgroup analysis according to screw loosening. The rate of bone nonunion was higher in the patients with screw loosening than in those without (41.7% vs. 5.4%, *p* < 0.001). In the patients without screw loosening (n = 224), the rate of patients with MF FI ≥ 25% in the nonunion group was higher than that in the union group but without significant difference (5.7% vs. 5%, *p* = 0.824). However, in the patients with screw loosening (n = 96), the nonunion group had a significantly higher rate of MF FI ≥ 25% than the union group (61.8% vs. 14.6%, *p* < 0.001).

**Table 4:** Comparisons of Complications at 1-Year Follow-Up After Case–Control Matching

Variable	FI > 25% (n = 160)	FI < 25% (n = 160)	*P* value
Bone nonunion (yes)	40	12	< 0.001**
Screw loosening (yes)	55	41	0.073

The numbers in bold represented that there was significant difference between the two groups

**P < 0.01

## Discussion

In this study, we found that bone nonunion had a significantly higher risk to occur in FI ≥ 25% group. In accordance with the present results, previous studies have also demonstrated that as fat deposit of paraspinal muscles increased, the incidence of bone nonunion increased [21, 22]. Besides, Choi et al. [23] indicated that muscle cross-sectional area, another indicator of paraspinals degeneration, was also associated with bone nonunion. These findings might corroborate the idea that the degeneration of MF plays an important role in bone union. MF, as the most medial and largest of the paraspinal muscles, can provide most stability to the lumbar segments. Therefore, FI could influence the normal functions of MF and thereby deteriorate the process of bone union [24]. On the other part, previous study revealed significant correlations between FI of paraspinals and vertebral bone marrow fat content [25]. Because osteoblasts and adipocytes share a common precursor in the bone marrow, increased adipogenesis might be associated with decreased osteoblastogenesis [26, 27]. Another hypothesis suggested that high paraspinals FI were detrimental for bone union as it might reduce vascular ingrowth into fusion mass [28].

Further, we found that high FI would affect the bone union in the patients with screw loosening, whereas FI did not have such effect once patients did not occur screw loosening. It suggested that high MF FI might play a secondary role in the bone nonunion. In patients with unsatisfied screw fixation, low MF FI could be a protective factor of bone nonunion, which indicated that a rehabilitation training of paraspinal muscles was instrumental [29]. Previously, Lee et al. [21] quantified the fat content using a subjective semiquantitative scale and proposed that above grade 1 of FI at surgery was a risk factor. Our finding, while preliminary, suggested that 25% could be an available cutoff value to distinguish high-risk group for bone nonunion.

In light of screw loosening, the relationship between MF FI and screw loosening was still controversial. In our study, the incidence of screw loosening was higher in FI ≥ 25% group than in FI < 25% group , but without significant difference. This outcome was contrary to that of Kim et al. who reported that greater FI of MF had significant effects on the S1 screw loosening in 156 patients with degenerative lumbar diseases [30]. In contrast, a retrospective study of 137 degenerative lumbar scoliosis (DLS) patients by Leng et al. [16] demonstrated that FI of MF was irrelevant to screw loosening in corrective surgery, which was consistent with our study. A possible explanation for this might be that the degeneration of paraspinal muscles can affect screw loosening observably only in long level fusion. Leng et al.’s study might support this speculation. They found that degeneration of psoas muscles and erector spinae could affect screw loosening in six- or more-level fusion in corrective surgery for DLS, whereas the four- or five-level fusion had no this influence [16].

Our findings also revealed that the MF FI was not related to postoperative improvement of function status and symptoms of LSS patients. These results were in line with those of previous studies [31]. In a prospective multicenter cohort study, Betz et al. [31] found that fatty degeneration had rarely prognostic value of improvement in symptoms in LSS treatment. Similarly, Bhadresha et al. [32] reported that there was a tendency toward greater improvements between baseline and 12-month follow-up in patients with Goutallier stage 1 or lesser (low FI), but these improvements did not differ significantly. However, contrary findings also exist in the current research [13, 33]. A study based on MRI and 2-year follow-up of patients from Kjersti et al. demonstrated that lower pre-treatment fat infiltration rate predicted greater improvement of pain and ODI [33]. In addition, Wang et al. [13] indicated that preoperative MF FI was significantly correlated with postoperative ODI and ODI improvement. This discrepancy might be attributed to diverse diseases, surgical methods and follow-up durations in studies.

In general, our results could not support the hypothesis of association between FI and the improvement of symptoms. In a community-based study, they also found no association between paraspinal muscles density and the occurrence of low back pain [34]. A possible explanation for this might be that FI may just play a small part in pain. Several muscular reasons including lower muscle strength, muscle atrophy, FI and inflammatory may act together on the occurrence of pain [35].

## Limitations

There are some limitations in this study. Firstly, this study was a retrospective study, which might cause the bias. But 320 subjects after case–control matching were included in our study, whose sample size was larger than previous studies. Besides, the methods to distinguish the adipose tissue vary in different studies, leading to a deviation of FI [16], while we applied the threshold technique which can automatically differentiate fat from lean muscles and has a good reliability.

## Conclusions

In conclusion, bone nonunion had a significantly higher rate to occur in FI ≥ 25% group. However, there were no differences in the incidence of screw loosening and postoperative improvement in functions and symptoms between FI ≥ 25% and FI < 25% groups.

## Supplementary Information

**Additional file 1**: **Figure S1.** Representative patients. Fig (a), a 52-year-old man with MF FI < 25% before surgery. Fig (b), a 54-year-old woman with MF FI > 25% before surgery.

## Data Availability

The data used and analyzed during the current study were available from the corresponding author on reasonable request.

## Abbreviations

BMI: Body mass index
CCM: Case-control matching
CSA: Cross-sectional area
CSI: Clinically significant improvement
CT: Computed tomography
DLS: Degenerative lumbar scoliosis
FI: Fat infiltration;
LSS: Lumbar spinal stenosis
MF: Multifidus
MRI: Magnetic resonance imaging
ODI: Oswestry Disability Index
VAS: Visual analog scale
