# Supplementary figures and images for: Can fat infiltration in the multifidus muscle be a predictor of postoperative symptoms and complications in patients undergoing lumbar fusion for degenerative lumbar spinal stenosis? A case–control study
Source: J Orthop Surg Res. 2022 May 26;17:289. doi: 10.1186/s13018-022-03186-2 (PMC9137055; doi:10.1186/s13018-022-03186-2)

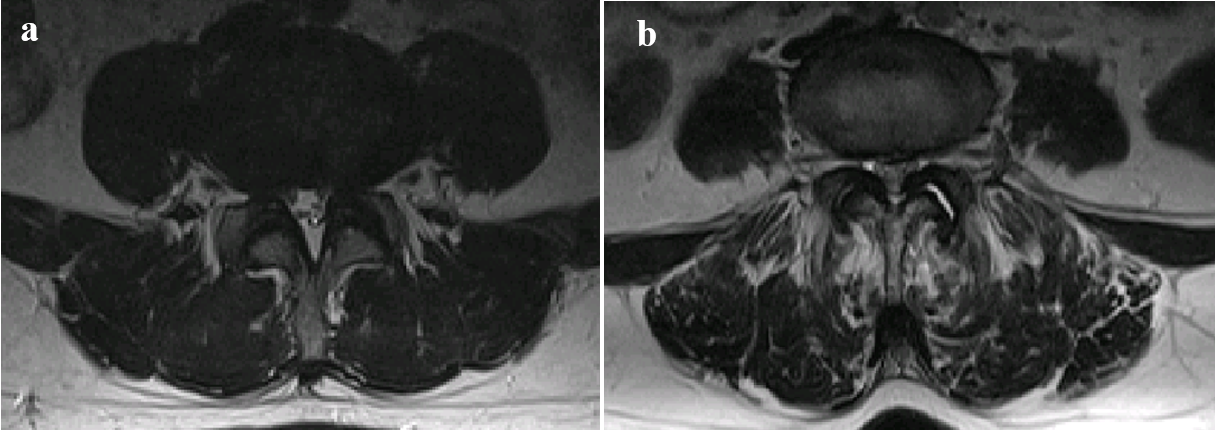

Supplement: Supplementary file 1 — Additional file 1: Figure S1. Representative patients. Fig (a), a 52-year-old man with MF FI < 25% before surgery. Fig (b), a 54-year-old woman with MF FI > 25% before surgery. [file 13018_2022_3186_MOESM1_ESM.png]
